# Midazolam sedation in palliative medicine: retrospective study in a French center for cancer control

**DOI:** 10.1186/s12904-020-00592-3

**Published:** 2020-06-19

**Authors:** Vincent Gamblin, Vincent Berry, Emmanuelle Tresch-Bruneel, Michel Reich, Arlette Da Silva, Stéphanie Villet, Nicolas Penel, Chloé Prod’Homme

**Affiliations:** 1 Palliative care unit, Oscar Lambret center, 3 rue Frédéric Combemale, 59020 Lille, France; 2 Palliative care unit, Maison Médicale Jean XXIII, 3 Place Erasme de Rotterdam, 59160 Lille, France; 3 Direction of Research and Innovation, Oscar Lambret center, 3 rue Frédéric Combemale, 59020 Lille, France; 4 Lille University Hospital and Medical School, 59000 Lille, France; 5 Lille University Hospital and Medical School, Palliative care unit, 59000 Lille, France; 6 ETHICS (Experiment, Transhumanism, Human Interactions, Care and Society) – EA 7446, Lille Catholic University, 59800 Lille, France

**Keywords:** Sedation, Midazolam, Palliative care, Oncology

## Abstract

**Background:**

French legislation about sedation in palliative medicine evolved in 2016 with the introduction of a right to deep and continuous sedation, maintained until death. The objective was to describe midazolam sedation at the COL (*Centre Oscar Lambret* [Oscar Lambret Center], French regional center for cancer control), in order to establish a current overview before the final legislative changes.

**Methods:**

Descriptive, retrospective and single-center study, concerning major patients in palliative care hospitalized from 01/01/2014 to 12/31/2015, who had been sedated by midazolam. The proven sedations (explicitly named) and the probable sedations were distinguished.

**Results:**

A total of 54 sedations were identified (48 proven, 6 probable). Refractory symptoms accounted for 48.1% of indications, complications with immediate risk of death 46.3%, existential suffering 5.6%. Titration was performed in 44.4% of cases. Sedation was continuous until death for 98.1% of the cases. Probable sedation had a higher failure rate than proven sedation. Significant differences existed for the palliative care unit compared to other units regarding information to the patient, their consent, anticipation, mention by correspondence and carrying out titrations. When patients had already been treated with midazolam, the induction doses, initial maintenance doses, and doses at the time of death were significantly higher. For those receiving opioids, the maintenance dose at the time of death was higher. No comparison found a difference in overall survival.

**Conclusions:**

After a sufficient follow-up has enabled teams to familiarize with this new legislation, reflection on sedation should be conducted to adapt to final recommendations.

## Background

Despite numerous national and international recommendations [1–5], sedation in palliative medicine remains a complex practice and a source of questioning that generates ethical, legal and societal discussions [6, 7]. Deep and continuous sedation, already practiced in several countries, is particularly controversial [8, 9]. Sedation in the palliative or terminal phase has been practiced by many teams for several years, but French legislation has recently changed, with law no. 2016–87 of February 2nd, 2016 creating new rights for those facing illness or death [10], (*Claeys-Leonetti law*), establishing a right to deep and continuous sedation until death at the request of the patient.

The law of February 2nd, 2016 also insists on seeking the patient’s consent, directly or through advance directives, on the place of relatives and especially the person of trust, and on the collegiate procedure in the decision to implement the sedation.

The Oscar Lambret Center (COL) is cancer center for the Hauts-de-France region in the north of France. Each year it treats 7000 patients with solid tumors. It employs 800 people including 120 doctors and has 180 beds. The COL has 4 oncology medical departments (with a total of 48 beds) and an 11-bed palliative care unit (PCU). The care of patients at the end of their life is not restricted to PCUs, and midazolam sedation is a practice implemented by most care teams. In addition, a mobile intra-hospital palliative care unit provides daily in-patient consultations.

The aim of this work was to describe the midazolam sedation practices at the COL for palliative care patients, in order to establish a current overview before the 2016 legislative changes, whose clinical recommendations by the HAS (*Haute Autorité de Santé* [High Authority for Health]) were published in 2018.

## Methods

### Study objective and design

It was a descriptive, retrospective and single-center study, based on a series of cases. The main objective was to describe midazolam sedation practices in a context of palliative care in COL. Secondary objectives were to compare the practices according to the medical supervision unit, the activity period, the indication of sedation, and to assess the effect of prior medication on midazolam dosages and overall survival.

### Inclusion and exclusion criteria

The population concerned the adult patients in palliative care hospitalized at the COL from 01/01/2014 to 12/31/2015, who had been sedated by midazolam. Patients prescribed midazolam were identified using data from the PMSI (a tool for describing hospital activities and measuring their cost) provided by the COL’s Medical Information Department. The palliative stage of care was sought in the report of the multidisciplinary team meeting, attended by oncologists, radiation therapists and surgeons. Following an initial evaluation, the two-year period of analysis was decided on based on the estimated number of 300 patients per year hospitalized for palliative care, with an estimated rate of sedation by midazolam of 10% representing 60 patients over two years. With such a sample size, the precision of the bilateral 95% confidence interval would be +/− 13% for an estimated rate of sedation for refractory symptom of 50%.

**Fig. 1:**
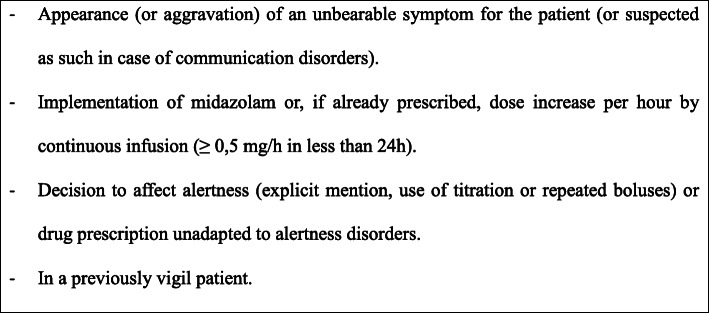
Criteria of « probable sedation »

The research in midazolam prescriptions was carried out thanks to the computerized patient file. Sedation was considered “proven” if explicitly mentioned in the outgoing correspondence and/or in the daily observations. We felt that the explicit use of the term “sedation” in the medical comments reflected the prescriber’s intentions, regardless of how this sedation was implemented. In its absence, the file was deemed as “probable sedation,” subject to validating several criteria (Fig. 1). In these situations, as the specific intentions of the prescriber were not known as they were not stipulated, we felt that these objectifiable criteria best reflected the prescriber’s true intention, by definition subjective and difficult to access. Lastly, in the case of several sedations for a same patient, only the first sedation was taken into account.

In cases of sedation for psychological or existential suffering, the patient is evaluated by the psycho-oncology team (psychiatrists and psychologists) alongside the overall assessment by the care team. Different assessment tools are used, such as the Hospital Anxiety and Depression Scale.

### Data collected

The following elements were identified in the medical prescription software DxCare©: general characteristics of patients, context of sedation, information given and its traceability, decision-making procedures, sedation implementation measures, follow-up and end of sedation.

### Statistical methodology

Characteristics of patients and sedation practices were described using frequencies, percentages, 95% confidence intervals (95% CI), means, standard deviation (+/−), median and range. Groups of patients with proven and probable sedation were compared to ensure the population could be jointly analyzed, and all subsequent analysis were done firstly on overall population, then on the subgroup of patients with proven sedation as sensitivity analysis. Characteristics of sedation practices were compared between PCU and other units, between on-call duty and normal activity periods, and according to the indication of sedation. Midazolam dosages at induction dose, maintenance dose and at the time of death were compared according to prior exposure to midazolam, to strong opioids and to medication affecting alertness. Comparisons of categorical variables between groups were performed using Khi-2 test or Fisher exact test in the case of small counts. Continuous variables were compared using Student t test if applicable or Wilcoxon Mann-Whitney test otherwise. Overall survival, defined as time interval from sedation to death, was estimated using Kaplan-Meier method and was compared between groups using Logrank-test. Significance level was set to *p* < 0.05. The data was extracted from the medical prescription software DxCare© and the electronic medical record SICOL©, the results were analyzed by the two main authors and a biostatistician. Statistical analyses were performed using Stata 13.1 (StataCorp. 2013. Stata Statistical Software: Release 13. College Station, TX: StataCorp LP).

### Legal requirements for collecting personal data

As the center had already informed the French Data Protection Authority that it keeps computerized files, no additional declaration was necessary.

## Results

### Population

Over this 24-month period, there were 601 adult patients hospitalized for palliative care, and 512 deaths. The study population consisted of 54 patients, i.e. 48 confirmed and 6 probable sedations. Midazolam sedation involved 9.0% (95%CI: 6.8–11.6%) of palliative patients and 10.5% (95%CI: 8.0–13.5%) of deceased patients.

There were 31 women and 23 men, with a mean age of 56.9 years (+/− 13.1). All sedations were performed in the wards of conventional medicine hospitalizations (none in surgery). PCU was the most frequent sedative service (31.4%). Breast (20.4%), upper digestive tracts (18.5%) and bronchial (11.1%) cancers were the most common primary tumors (Table 1). The indications for sedation consisted mainly of refractory symptoms (48.1, 95%CI: 34.3–62.2%) and immediate life-threatening complications (46.3, 95%CI: 32.6–60.4%) as asphyxia-type respiratory distress or major blood loss. Three cases of sedation for psychological or existential suffering were found (5.6%). The main symptoms were: respiratory disorders (51.8%), agitation/confusion (16.7%), hemorrhage (11.1%).

**Table 1:** Patients general characteristics and indication for sedation

Characteristics (***N*** = 54)	n	%
**Age (years)**		
Median (range)	56.9	(25.6–85.7)
Mean (standard deviation)	56.9	(13.1)
**Gender**		
Female	31	57.4%
Male	23	42.6%
**Medical supervision unit**		
Palliative care	17	31.5%
Uro-digestive	12	22.2%
Breast	10	18.5%
General Oncology	8	14.8%
Upper aerodigestive tract	5	9.3%
Gynecology	2	3.7%
**Primitive Tumor**		
Breast	11	20.4%
Upper aerodigestive tract	10	18.5%
Lung	6	11.1%
Colon-rectum	4	7.4%
Œsophagus	4	7.4%
Soft tissue sarcoma	4	7.4%
Cervix	2	3.7%
Prostate	2	3.7%
Bladder	2	3.7%
Anal canal	1	1.9%
Lymphoma	1	1.9%
Bone	1	1.9%
Ovary	1	1.9%
Skin	1	1.9%
Kidney	1	1.9%
Biliary	1	1.9%
Unknown	2	3.7%
**Indication for sedation**		
Immediate risk of death	25	46.3%
*Respiratory distress*	*18*	*33.3%*
*Hemorrhage*	*5*	*9.3%*
*Hemodynamic shock*	*2*	*3.7%*
Refractory symptoms ^a^	26	48.1%
*Respiratory disorders*	*10*	*18.5%*
*Agitation and confusion*	*9*	*16.7%*
*Anxiety*	*4*	*7.4%*
*Pain*	*2*	*3.7%*
*Bleeding*	*1*	*1.9%*
*Asthenia*	*1*	*1.9%*
*Status epilepticus*	*1*	*1.9%*
Existential suffering	3	5.6%

^a^ Some patients had several symptoms

### Sedation practices

The mention of information given to the patient was present in 40.7% of cases, and to his relatives in 72.2%. Patient consent was mentioned in 31.5% of the cases. The collegiate nature of the decision (involving at least 2 doctors) appeared in 37.0% of the cases. An early prescription was made for 27.8% of the sedations. 68.5% of the end-of-hospitalization communication reported sedation. For all cases of probable sedation (11.1%), no written mention was found.

Midazolam was systematically administered intravenously. All but one of the sedations were prescribed for indefinite periods. Induction was performed in 48.1% of cases, with true titration for 44.4%. The average dose for bolus was thus 1.6 mg +/− 2.1, the total average dose administered during titration (until sedation) was 3.8 mg +/− 3.1. The initial maintenance dose (either from the outset or after titration) averaged 1.5 mg/h +/− 1.5. At the end of the sedation, the average flow rate was 3.6 mg/h +/− 4.4.

Patient vigilance was not assessed precisely, with no standard scale applied to almost all cases (Glasgow score for one case, Rudkin score for two). However, subjective assessments of patient relief were found. Forty-eight patients could be categorized into three categories: relieved (78.3%), partially relieved (6.5%), unrelieved (15.2%). It was not possible to conclude on 8 cases. In 11 situations (20.4%), a second sedation, defined by an explicit mention or new titration during sedation, was used (10 using midazolam and one using propofol).

All patients were on continuous infusion of midazolam at the time of death. The prognosis was quickly poor, with a median overall survival of 1 day (95% CI: 1–2 days, range: 0–20 days). The 5-day overall survival rate was 5.7% (95% CI: 1.5–14.2%).

There was no significant difference between proven and probable sedation, excepted regarding the relief of the patient with no patient appeased after probable sedation (*p < 0.001*) (Table 2).

**Table 2:** Characteristics according to proven or probable sedation

Characteristics	Proven sedation (***N*** = 48)		Probable sedation (***N*** = 6)		***p***-value
**Indication**					
Immediate risk of death	21	43.8%	4	66.7%	0.60
Refractory symptom	24	50.0%	2	33.3%	
Existential suffering	3	6.3%	0	0.0%	
**Relief of the patient**					
Unrelieved	3	7.1%	4	100.0%	< 0.001
Relieved	36	85.7%	0	0.0%	
Partially relieved	3	7.1%	0	0.0%	
Unknown	6		2		
**Information of the patient**	21	43.8%	1	16.7%	0.38
**Information of the family**	35	72.9%	4	66.7%	1.00
**Consent of the patient**	16	33.3%	1	16.7%	0.65
**Collegiality**	18	37.5%	2	33.3%	1.00
**On-call duty**	27	56.3%	3	50.0%	1.00
**Second sedation**	10	20.8%	1	16.7%	1.00
**Continuous sedation**	48	100.0%	5	83.3%	0.11
**Titration**	23	47.9%	1	16.7%	0.21
**Early prescription**	14	29.2%	1	16.7%	1.00
**Associated opioids**	44	91.7%	5	83.3%	0.46
**Induction dose (mg)**	N = 25		*N* = 1		ND^a^
Median (range)	3.0	(0.5–10)	3.5		
Mean (sd)	3.9	(3.2)			
**Maintenance dose (mg/h)**	N = 48		N = 6		0.68
Median (range)	1.0	(0.2–7)	1.0	(0.2–2)	
Mean (sd)	1.5	(1.5)	1.1	(0.8)	
**Dose at the time of death (mg/h)**	*N* = 47		N = 6		0.21
Median (range)	2.0	(0.2–24)	3.0	(2.5–20)	
Mean (sd)	3.4	(4.0)	5.7	(7.0)	

^a^ND: not done (poor sample size)

### Comparisons of sedation practices

Within the PCU, the proportion was greater for cases where the information given to the patient was reported and with consent (Table 3). Sedation was more often reported in the end-of-hospitalization correspondence. Sedation prescriptions in the PCU were more frequently in advance, and the proportion of titration was higher. The same significant differences were found when only considering proven sedations. In this population, the mention of information given to the family was higher in the PCU (15/16:93.8% vs. 20/32:62.5%, *p = 0.036*) whereas significance was not reached in the overall population.

**Table 3:** Sedation practices according to medical unit and indication

Characteristics	Medical unit					Indication				
	Other unit (***N*** = 37)		PCU (***N*** = 17)		***p***-value	Immediate risk of death (N = 25)		Refractory Symptom (N = 26)		***p***-value
**Indication**										
Immediate risk of death	19	51.4%	6	35.3%	0.27	–		–		–
Refractory symptom	17	45.9%	9	52.9%						
Existential suffering	1	2.7%	2	11.8%						
**Relief of the patient**										
Unrelieved	7	22.6%	0	0.0%	0.13	2	9.5%	5	22.7%	0.48
Relieved	22	71.0%	14	93.3%		18	85.7%	15	68.2%	
Partially relieved	2	6.5%	1	6.7%		1	4.8%	2	9.1%	
Unknown	6		2			4		4		
**Information of the patient**	11	29.7%	11	64.7%	0.015	12	48.0%	7	26.9%	0.12
**Information of the family**	24	64.9%	15	88.2%	0.11	20	80.0%	16	61.5%	0.15
**Consent of the patient**	8	21.6%	9	52.9%	0.021	9	36.0%	5	19.2%	0.18
**Collegiality**	11	29.7%	9	52.9%	0.10	9	36.0%	8	30.8%	0.69
**On-call duty**	20	54.1%	10	58.8%	0.74	15	60.0%	15	57.7%	0.87
**Second sedation**	7	18.9%	4	23.5%	0.73	5	20.0%	5	19.2%	1.00
**Correspondence**	21	56.8%	16	94.1%	0.006	17	68.0%	17	65.4%	0.84
**Probable sedation**	5	13.5%	1	5.9%	0.65	4	16.0%	2	7.7%	0.42
**Continuous sedation**	36	97.3%	17	100.0%	1.00	25	100.0%	25	96.2%	1.00
**Titration**	12	32.4%	12	70.6%	0.009	10	40.0%	12	46.2%	0.66
**Early prescription**	5	13.5%	10	58.8%	0.001	6	24.0%	9	34.6%	0.41
**Associated opioids**	32	86.5%	17	100.0%	0.17	23	92.0%	23	88.5%	1.00
**Induction dose (mg)**	*N* = 14		*N* = 12		0.74	*N* = 10		*N* = 13		0.35
Median (range)	3.0	(0.5–10)	3.0	(0.5–10)		2.5	(0.5–8)	3.0	(0.5–10)	
Mean (sd)	3.8	(3.3)	4.0	(3)		2.9	(2.2)	4.5	(3.8)	
**Maintenance dose (mg/h)**	N = 37		N = 17		0.20	N = 25		N = 26		0.22
Median (range)	1	(0.2–7)	1.2	(0.2–5)		0.6	(0.2–7)	1.1	(0.2–5)	
Mean (sd)	1.3	(1.4)	1.8	(1.5)		1.2	(1.5)	1.7	(1.5)	
**Dose at the time** **of death (mg/h)**	*N* = 36		N = 17		0.86	*N* = 24		N = 26		0.27
Median (range)	2.5	(0.2–20)	2.0	(0.4–24)		3.0	(0.2–20)	2.0	(0.4–10)	
Mean (sd)	3.5	(3.7)	4.0	(5.7)		3.8	(4.2)	2.7	(2.5)	

The sedations performed during the periods of on-call duty accounted for 55.6% of the cases (*n* = 30), with no significant difference found with the sedations started during periods of “normal service”, in overall population and in proven sedations.

Due to the small number of psychological or existential sufferings (n = 3), the comparison was made only between refractory symptoms (*n* = 26) and immediate risk of death complications (*n* = 25). No significant difference was found. In a non-significant way, providing information to the patient and his relatives was more frequent for complications at immediate risk of death. The significance threshold was reached only for proven sedation (respectively 57.1% vs. 25.0%, *p = 0.028* and 85.7% vs. 58.3%, *p = 0.043*).

### Impact of prior medication on midazolam dosage

A total of 68.5% of patients had received midazolam prior to sedation. In the case of prior exposure, the dosages used during sedation were higher for the maintenance dose as for the dose at the time of death. In case of induction, the dose was significantly higher (Table 4).

**Table 4:** Dose of midazolam according to prior medications

Characteristics	Induction dose (mg)						Maintenance dose (mg/h)						Dose at time of death (mg/h)					
	N	Median (range)		Mean (sd)		*p*-value	N	Median (range)		Mean (sd)		*p*-value	N	Median (range)		Mean (sd)		*p*-value
**Prior exposure to midazolam**																		
No (N = 17)	8	1.5	(0.5–3.0)	1.6	(1.1)	0.004	17	0.4	(0.2–1.5)	0.5	(0.4)	< 0.001	16	1.0	(0.2–8.0)	1.8	(2.0)	0.003
Yes (N = 37)	18	3.8	(1.0–10.0)	4.9	(3.2)		37	1.5	(0.5–7.0)	1.9	(1.6)		37	3.0	(0.6–24)	4.4	(4.9)	
**Prior exposure to strong opioids**																		
No (*N* = 7)	4	2.0	(1.0–3.0)	2.0	(0.8)	0.22	7	0.6	(0.2–1.5)	0.8	(0.4)	0.31	7	1.0	(0.5–3.0)	1.3	(0.8)	0.025
Yes (N = 47)	22	3.0	(0.5–10)	4.2	(3.3)		47	1.0	(0.2–7.0)	1.6	(1.6)		46	2.8	(0.2–24)	4.0	(4.6)	
**Prior exposure to medications** **that impair alertness**																		
No (*N* = 33)	15	3.0	(0.5–10.0)	3.8	(3.2)	0.81	33	1.0	(0.2–7.0)	1.4	(1.5)	0.46	33	2.0	(0.2–9.0)	2.7	(2.4)	0.18
Yes (*N* = 21)	11	3.0	(0.5–10.0)	3.9	(3.2)		21	1.0	(0.2–5.0)	1.6	(1.4)		20	2.8	(0.5–24)	5.1	(6.3)	

The associated prescription for strong opioids was 87.0%. The maintenance dose at the time of death was significantly higher for this patient population.

A total of 38.9% of patients had received medication affecting alertness within 24 h of sedation. These drugs were benzodiazepines (other than midazolam, 22.2%), amitriptyline (12.9%), scopolamine (11.1%), antipsychotics (11.1%), hydroxyzine (7.4%), and ketamine (7.4%). There was no significant difference in midazolam dosage when co-administered with one or more of these drugs.

## Discussion

The findings of this study make it possible to better understand what are relatively rare sedation practices for patients hospitalized in cancer centers. Most indications are for refractory symptoms and acute complications that are immediately life-threatening, each accounting for almost 50% of cases. Sedation was maintained until the patient’s death in almost all cases (98%). In most cases sedation was implemented in accordance with HAS guidelines, with the exception of the low frequency of titration. Information provided to the loved ones, the collegiality of the decision making, and the traceability of the sedation were insufficient.

### What this study adds

The prevalence of sedation in our study is lower than that found in the literature (14.6 to 66.7% in the review of Maltoni et al. [11]), but these frequencies vary in large proportions because of differences between the definitions, indications and practices selected according to the studies [11, 12]. These findings are nevertheless in line with the opinion of French experts, who consider that situations possibly warranting sedation are relatively rare [13].

The most frequent indications in the litterature for sedation are delirium and dyspnea. While pain is scarcer in this regard [11, 12, 14–26], it is a frequent reason for hospitalization in the COL. The indication for dyspnea was, however, predominant in the COL. There was also a significant proportion of indications related to hemorrhages (11.1% vis-a-vis 1.6 to 3.3% in the work of Benitez-Rosario [16]). The differences can be explained by the fact that our study took into account all indications of sedation: while the published works are generally limited to deep and continuous sedations for refractory symptoms, our findings show that sedation is frequently used for acute complications that are immediately life-threatening. These indication is distinguished only by the SFAP (*Société française d’accompagnement et de soins palliatifs* [French society for end-of-life and palliative care]) [1] and does not exist in the other recommendations [3–5]. Such complications often involve terminal hemorrhage and asphyxia-type respiratory distress. The purpose of sedation is to relieve the patient of the panic and terror that these situations cause, and which are also generally very stressful for the family and caregivers. In the terminal phase, sedation is an emergency procedure that can affect the time of death. Moreover, the frequency of information provided to patient and family was greater for immediate life-threatening complications than for refractory symptoms, these differences becoming significant only when known sedations were considered. The severity of these situations as well as the likelihood of a very rapid death may have prompted professionals to devote more time to their care.

The proportion of titrations was low (44.4%). In the majority of cases, the midazolam maintenance dose was directly introduced or increased arbitrarily and then adjusted in stages. With the exception of those of the SFAP [1], no recommendation specifically describes the titration procedures, and many studies do not detail or explicitly use them [11, 12, 14–24, 26–34]. However, the absence of titration exposes to risks of over or under dosages, because of a large inter-individual variability of the sensitivity to midazolam [35, 36], as well as to a delay in relief [35, 36]. Titration also has an important ethical value, demonstrating the principle of proportionality [37]. This principle, by the use of adapted doses in order to obtain sedation, allows to differentiate between sedation and euthanasia, among other criteria.

In addition, our findings reveal two other arguments that titration is essential, whetherin routine clinical practice or in clinical studies.

The frequent absence of titration may explain the use of higher doses: 36 mg/d at the beginning of the sedation and 86.4 mg/d at the end in our study, whereas the usual reported doses are between 10 mg/d et 50 mg/d [15, 16, 18, 21, 38], more rarely beyond this (up to 75 mg/d in the Caraceni study [34]). Prior administration of midazolam was associated with higher doses of midazolam to initiate and maintain sedation as well as at the time of death. This observation is explained by the phenomenon of tachyphylaxis [18, 36], but its physiopathology is not fully explained, and several mechanisms have been reported [35, 36, 39].

Titration makes it possible to search for the minimum effective dosage, in the context of sedation that is proportionate to the severity of the symptoms, the objective being to reduce or eliminate the patient’s exposure to an unbearable situation, without necessarily becoming completely unconscious.

The information of the patient and the research of his consent were found to be inconsistent (respectively 40.7 and 31.5%). These frequencies are similar in several studies [10–22, 24, 26–28]. Patients at the end of life often have impaired alertness, rendering the obtaining of consent impossible or inappropriate. The proportion of patients that was able to express an opinion about sedation in a palliative setting was about 50% in 2 published studies [20, 26]. But even in the absence of an impairment of judgment, consenting to sedation remains a difficult decision for patients.

Notification was given to the family in a greater proportion (72.2%). However, it is often close to 90% in many publications [12, 16, 19, 22, 26, 29]. This may be explained by a lack of information, a lack of traceability, or even insufficient foresight, as suggested by the low rate of anticipated prescriptions of 27.8%. The time to talk with loved ones was then probably shorter, hence more succinct and less established information.

The collegiality of the decision-making process was quite rare (37.0%). The frequencies of collegial meetings reported in the literature are higher, between 54 and 70% [19, 23, 30]. The frequent participation of COL doctors in oncological multidisciplinary consultation meetings could lead to confusion between the decision for palliative care and that of sedation. It is also important to remember that for deep and continuous sedation maintained until death, the French law requires a meeting to ensure a collective procedure, but such meetings are simply recommended for other sedation practices. All the arguments put forward during this meeting and proof that the patient’s consent has been sought must be entered in the patient’s file [10].

The sedations performed in the PCU were more often associated with information provided to the patient and the patient’s consent, and even more frequent information provided to the family when probable sedations were excluded. Anticipation of sedation was more common in the PCU. The use of an initial titration was also predominant in the PCU. The palliative care team had a priori better training and greater experience in sedation. Daily collegial meetings probably facilitated the exchange of information and identification of risk situations. Our results therefore confirm those of a study in the Netherlands in 2007 [40]**,** describing greater compliance with sedation recommendations when the prescribing doctor specializes in palliative care.

All cases of sedation for psychological or existential suffering included the trace of a discussion with the patient, his/her consent, as well as providing information to the relatives. Collegiality was systematically found, and the carrying out of sedation was reported in the end-of-hospitalization communication. It can be assumed that the complexity of these situations, the reflection and the time required for the decision have favored a richer discussion between the team, the patient and his family, as well as a better traceability. Deep and continuous sedations for psychological or existential refractory suffering is subject of discussion and needs to be clarified because no consensus is yet available within medical societies [41]. When there is refractory psychological or existential suffering, the main goal of the psycho-oncology team is to look for differential diagnoses, such as depressive syndrome, demoralization, a desire to hasten death or a request for euthanasia [42].

The main limitation of this work was its retrospective nature, with a risk of poor traceability and therefore an underestimation of the practices studied; and low standardization of the declarative data that required interpretation or recoding. These mainly impacted decision-making and information provided, and prevented the depth of sedation from being collected. The impact of this bias on the study of the prescriptions was more limited, as their recording was automated by the software.

This was a single center study conducted in the specific context of palliative cancer care in the north of France. It may therefore be difficult to extrapolate our findings to other healthcare fields and systems.

### Perspectives

Our work involved identifying a certain number of probable sedation scenarios, corresponding to criteria very similar to routine sedation practices, but without being identified as such. The absence of any significant difference in their indications, methods and dosages suggested that they were indeed cases of sedation that fell within the scope of this study: it seemed unlikely that these prescriptions had been decided on without the prescriber being aware of the resulting decrease in alertness. However, the lack of an explicit appointment of sedation, its inefficiency and the more scarce information provided to the patient and his family made it seem less likely that the team would carry out these probable sedations, a confusion between implementation and result (carrying out sedation and managing to “sedate” the patient), a confusion in intention between anxiolysis and sedation, or a concealment of failure. However, given the low number of probable sedations, the sensitivity was poor. Assessment of comfort was not available for a significant proportion of patients (8/54), which could also bias the analysis. In a Canadian study of medication prescriptions used for sedation, 64.5% of cases had no explicit mention [21].

The identification of these probable sedation cases illustrates the need for further training for care teams on this complex practice.

The French society for assistance and palliative care (SFAP) recently created a tool called SEDAPALL [43] to describe and analyze end-of-life palliative sedation practices. It is both an educational tool and a research tool that helps to ascertain the intentions behind the decision. It makes it possible to verify whether or not the intention is actually put into effect. The intentionality of sedation is described by SEDAPALL based on 3 criteria:

- Duration: transient, indeterminate or maintained until death.
- Depth: proportionate or deep from the outset.
- Level of consent: not obtained, obtained in advance, obtained at the onset of sedation, requested by the patient.

A sedation working group has been set up for the C3 - the three cancer centers in North West France (Caen, Rouen and Lille). Based on the findings of our study, which will illustrate sedation practices in cancer centers, and given that it is vital to characterize probable sedation scenarios, the objective of this working group will be to develop an educational activity regarding sedation practices to enable medical and healthcare teams to implement the SEDAPALL tool and to reflect on their daily clinical practices.

## Conclusion

This study gives a better understanding of sedation practices for patients hospitalized in cancer centers for palliative care. It reveals the need for these facilities to meticulously characterize their sedation practices to correct deviations from good practice guidelines.

The risk of these gaps would be not to meet the ethical requirements that guide this practice.

The law of February 2nd, 2016 profoundly modify these practices and this work should therefore be updated after a sufficient follow-up has enabled teams to adapt to this new legislative context.

Several avenues can be explored to improve patient care. Further palliative care training should be provided to staff, especially to be able to use the SEDAPALL tool. The drafting of a procedure or creation of a form specifically on sedation at COL could encourage the teams to reflect more in their daily practice, but this should not turn into a checklist that does not take into account the particularities of each situation. The reflection and the ethical approach prior to sedation in the palliative phase are complex steps that cannot be limited to the application of a protocol. The multidisciplinarity should be improved, for example by using a palliative care resource team whenever possible, and we must ensure that care is anticipated.

In this way, improvements in palliative care sedation practices will help support patients as humanely as possible during the time they have left to live.

## Data Availability

The datasest used and analysed during the current study are available from the corresponding author on reasonable request.

## Abbreviations

COL: Centre Oscar Lambret (Oscar Lambret Center)
HAS: Haute Autorité de Santé (High Authority for Health)
PCU: Palliative Care Unit
PMSI: Programme de Médicalisation des Systèmes d’Information (Information Systems Medicalization Program)
SFAP: Société Française d’Accompagnement et de soins Palliatifs (French Society for Assistance and Palliative Care)
